# Performance during a 20-km cycling time-trial after caffeine ingestion

**DOI:** 10.1186/s12970-014-0045-8

**Published:** 2014-08-30

**Authors:** Henrique Bortolotti, Leandro Ricardo Altimari, Marcelo Vitor-Costa, Edilson Serpeloni Cyrino

**Affiliations:** 1 Metabolism, Nutrition, and Exercise Research Group, Londrina State University, Londrina, Paraná, Brazil; 2 Neuromuscular System and Exercise Research Group, Londrina State University, Londrina, Paraná, Brazil; 3 Department of Physical Education, Centre for Physical Education and Sport, Londrina State University, Londrina, Paraná, Brazil; 4 School of Physical Education and Sport, University of São Paulo, São Paulo, Brazil

**Keywords:** Ergogenic aid, Sports performance, Perceived exertion, Electromyography, Exercise testing

## Abstract

**Background:**

The objective of this study was to analyze the effect of caffeine ingestion on the performance and physiological variables associated with fatigue in 20-km cycling time trials.

**Methods:**

In a double-blind placebo-controlled crossover study, 13 male cyclists (26 ± 10 y, 71 ± 9 kg, 176 ± 6 cm) were randomized into 2 groups and received caffeine (CAF) capsules (6 mg.kg^−1^) or placebo (PLA) 60 min before performing 20-km time trials. Distance, speed, power, rpm, rating of perceived exertion (RPE), electromyography (EMG) of the quadriceps muscles and heart rate (HR) were continuously measured during the tests. In addition, BRUMS questionnaire was applied before and after the tests.

**Results:**

Significant interactions were found in power and speed (*P* = 0.001), which were significantly higher at the end of the test (final 2 km) after CAF condition. A main effect of time (*P* = 0.001) was observed for RPE and HR, which increased linearly until the end of exercise in both conditions. The time taken to complete the test was similar in both conditions (PLA = 2191 ± 158 s vs. CAF = 2181 ± 194 s, *P* = 0.61). No significant differences between CAF and PLA conditions were identified for speed, power, rpm, RPE, EMG, HR, and BRUMS (*P* > 0.05).

**Conclusion:**

The results suggest that caffeine intake 60 min before 20-km time trials has no effect on the performance or physiological responses of cyclists.

## Background

Recent studies have shown that caffeine (CAF) can act as an ergogenic aid, both in short and long-term exercise [1]–[4] at both central and peripheral level [4]–[6]. Conversely to what was initially thought, CAF intake does not seem to be able to accelerate fat metabolism and to spare muscle glycogen during exercise, which would explain the increased performance observed in endurance tasks [4],[7]. Currently, this potential effect of CAF is credited to its affinity to adenosine receptors (A_1_ and A_2a_). When CAF molecules bind with these pre and post synaptic receptors, it inhibits adenosine action, promoting the release of excitatory neurotransmitters, increasing corticomotor excitability [8],[9]. This stimulatory effect of CAF on the central nervous system may be responsible for modifying the motivation parameters that cause sustain discomfort during physical exercise, reducing the rating of perceived exertion (RPE) during exercise [10].

Although the ergogenic effect of CAF on the neuromuscular system has been discussed in detail in a previous review study [11], it is noteworthy that the majority of studies have so far adopted open-loop protocols. Despite being a sensitive test that quantifies changes in performance [12], it does not represent the reality of sports competitions. Although closed-loop protocols have been less frequently used in investigations on the effect of CAF on physical performance [13]–[16], they have greater ecological validity than open-loop protocols due to its similarity with actual competitive situations, as well as having the ability to evaluate athletes’ pacing strategy [17]. Moreover, few studies have investigated the effect of CAF on RPE on time trials, where the subject can choose and plan his pacing strategy during the effort. As a result, it has been difficult to extrapolate information on the use of CAF to competitive situations.

Therefore, the objective of the present study was to analyze the effect of CAF ingestion on the performance and physiological variables associated with fatigue in 20-km cycling time trials using a closed-loop protocol.

## Methods

### Experimental design

A double-blind, randomized, placebo-controlled crossover study with previous familiarization was approved by the Londrina State University Ethics Committee. Thirteen male cyclists (71 ± 9 kg; 176 ± 5 cm; 253 ± 142 km.week^−1^) with at least two years of competitive experience were recruited for the study. All participants had been free of injuries for at least six months before the tests. Prior to tests, the subjects visited the laboratory to become aware of the purpose of the study and sign an informed consent. Schedules were set, and subjects returned to the laboratory to perform anthropometric measurements and a pre-experimental trial to become familiarized with the equipment and the experimental protocol.

Participants were randomized into 2 groups and received caffeine (CAF) capsules (6 mg.kg^−1^) or placebo (PLA) 60 min before performing 20-km time trials in two different occasions separated by a minimum interval of 72 h. Therefore, the amount of CAF or PLA (maltodextrin) that the volunteers should ingest was determined from the body weight (i.e. a subject weighing 70 kg would ingest 420 mg of caffeine or placebo). Subjects were instructed to abstain from any CAF in the 48 h before the test. Furthermore, instructions were also given to abstain from alcohol intake and strenuous exercise in the 24 h prior to visiting the laboratory. For inclusion in the study, volunteers should not use other nutritional supplements. Ambient temperature and relative humidity in the laboratory were maintained between 21-24°C and 55-60%, respectively, in all tests. The subjects performed the tests always in the same period of the day to avoid the potential influence of circadian cycle.

During the time between ingesting the capsules and starting the test (60 min), the participants answered the Brunel mood scale (BRUMS) questionnaire, electrodes were placed, specific tests for EMG signal normalization were performed, and a 10-min warm-up was carried out.

### Pre-experimental test

Prior to the experimental tests, a maximal incremental test for determination of maximum parameters (power and HR) and physiological thresholds was performed, using specific software (Velotron CS 2008™ - RacerMate®, Seattle, WA, USA). After warming-up for 2 min at 100 W, the load was increased in 50 W at every 2 min until exhaustion or the inability to maintain the stipulated minimum cadence (70 rpm) for more than 5 s, despite verbal encouragement. The power reached in the last complete stage added to the product of the percentage of the time spent in the exhaustion stage by the standardized increment (50 W) was considered the maximum power (345.0 ± 41.6 W). The highest HR value at the last minute of test was recorded as the maximum HR (192 ± 11.6 bpm).

### Experimental protocol

Time trials were performed in a cyclosimulator (Velotron™ - RacerMate®, Seattle, WA, USA), which was calibrated prior to each test, according to manufacturer’s recommendations. The 20-km time trial was built in a straight line and 0° tilt using the same software used in the pre-experimental tests.

The subjects came to the laboratory on scheduled days and underwent a closed-loop test, in which they had to complete the 20-km time trial, in the shortest possible time with free choice of cadence and gear ratio, simulating an actual race. All participants received feedback on the time, power, RPM and distance traveled during the test on a monitor. Before, during and after the tests the following variables were analyzed: electromyographic activity of the muscles rectus femoris (RF), vastus medialis (VM) and vastus lateralis (VL), RPE, mood, and HR.

### Surface electromyography (EMG)

The torque-velocity test (T-V test) was performed to normalize the electromyographic activity [18]. After a 10-min warm-up at 100 W, each subject performed two maximum bouts with duration of 8 s each, with an interval of 5 min between bouts. The load during the test was 7.5% of the volunteer’s body mass. Participants were instructed to remain seated throughout the test. The electromyographic activity of each muscle was examined between the second and eighth seconds of each maximum bout, and the highest peak amplitude found, expressed in root mean square (RMS), was used as the normalization factor.

Electromyographic activity was monitored continuously during the tests in both experimental conditions (CAF or PLA) using an eight-channel electromyograph (TeleMyo 2400 T G2 - Noraxon Inc., USA). The sampling frequency for EMG records was 2000 Hz and the factor of common-mode rejection ratio was greater than 95 dB. The muscles examined were the superficial quadriceps femoris (QF), RF, VM and VL. The signal was recorded following the recommendations by ISEK. After site preparation by shaving, cleansing with alcohol and curettage to reduce skin impedance, active electrodes (TeleMyo 2400 - Noraxon Inc., USA) were fixed to the skin, with inter-electrode distance (center to center) of two centimeters. The reference electrode was positioned over the iliac crest. The location of the anatomical landmarks for electrode placement followed the standardization proposed by SENIAM [19].

### Analysis and processing of the EMG signal

RMS (μV) values were averaged for each 30-s period and were used for the analysis of electromyographic signals from RF, VM, and VL muscles and the integrated QF [(RF + VM + VL) / 3]. Data were processed using a mathematical simulation environment (Matlab 7.0 - MathWorks ®, South Natick, MA, USA). To obtain the values expressed in RMS, raw EMG signals were digitally filtered, using a band-pass filter of 20Hz and 500Hz, according to the procedures proposed by Dantas et al. [20].

### Measurement of perceived exertion

All subjects were instructed to report their perceived exertion according to the 6–20 point Borg scale [21] at each 2 km of exercise. From these data, we determined the intercept on the y axis (y-intercept), the coefficient of determination (R^2^) and the slope between the time and the individual perceived exertion values attributed during each test obtained by linear regression analysis.

### Psychological-motivational changes

On test days, subjects responded to the Brunel Mood Scale (BRUMS) when they arrived and after the experimental trial. This questionnaire was used for the detection of mood based on 24 questions, stratified into six areas, namely: confusion, anger, depression, fatigue, tension and vigor. Each domain score was normalized by the score obtained prior to the exercise by subtracting the scores at the end of the trial from the scores before the trial.

### Heart rate

During all testing protocols HR was monitored and recorded in RR intervals (ms) and beats per minute (bpm), using a heart rate monitor (Polar RS800CX - Polar®, Kempele, Finland). Data were recorded and stored for later beat-by-beat analysis of the heart’s R-wave signals through a coded Polar WearLink transmitter, positioned on the subject’s chest, allowing the transmission of data by telemetry.

### Statistical analyses

The normality of data was assessed by Shapiro-Wilk’s test. Levene’s test was used to analyze the homogeneity of variances. Two-way analysis of variance (ANOVA) for repeated measures was used for comparisons between conditions (CAF and PLA) and over time. The Bonferroni post hoc test was used when a significant F ratio was found for the main or interaction effect. A significance level of 5% was used for all analyzes.

Additionally, the practical inference based on magnitudes was also applied [22]. The chance of a given value to be beneficial (positive) or detrimental (negative) effect [e.g., higher or lower than the smallest worthwhile changes (0.20 multiplied by the initial standard deviation based on the effect size)] was calculated [23]. Thus, the change was assessed qualitatively as follows: <1% almost certainly not; 1-5% very unlikely, 5-25% unlikely, 25-75% possible, 75-95% likely, 95-99% very likely and > 99% almost certainly yes. When the negative and positive values showed results greater than 10%, the inference was considered inconclusive. The effect size (Cohen’s d) was also calculated for the time trial performance and interpreted using the recommendations suggested by Hopkins et al. [22] as follows: 0 = Trivial; 0.2 = Small; 0.6 = Moderate; 1.2 = Large; 2.0 = Very large; 4.0 = Nearly perfect.

## Results

**Figure 1:**
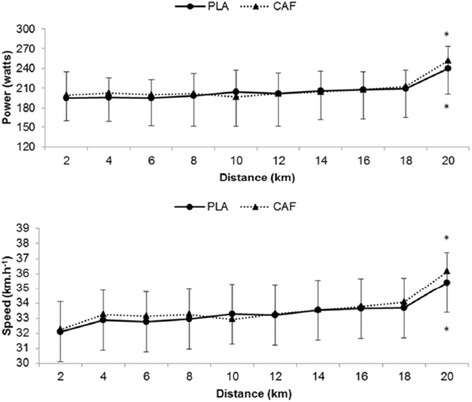
**Responses of power and speed on 20-km time-trial test under the conditions CAF (n = 13) and PLA (n = 13).** *P < 0.05 vs. 20 km. Significant main effect of time (*P* < 0.001).

Information on power, speed, pedaling cadence, HR and 20-km time trial test duration for PLA and CAF conditions are presented in Table 1. No significant differences were observed between CAF and PLA concerning HR and all the performance variables (*P* > 0.05). The results of the qualitative analysis proved inconclusive (unclear). The effect size was 0.06, being considered trivial. Power output and speed at every two kilometers in the 20-km time-trial, for CAF and PLA, are illustrated in Figure 1. Although a similar response was observed among groups (*P* > 0.05), a significant distance main effect in the last two kilometers of the test was observed with increased power and speed (*P* < 0.001). However, no significant group main effect or group by moment interaction was identified (*P* > 0.05).

**Table 1:** **Cycling performance indicators during the 20**-**km time trials**, **after acute ingestion of CAF** (**n** = **13**) **or PLA** (**n** = **13**). **Values are expressed as mean** ± **standard deviation**

	**Condition**		
Variables	PLA	CAF	*P*
Power (watts)	206.9 ± 28.5	204.6 ± 43.9	0.79
Speed (km.h^−1^)	33.5 ± 1.8	33.3 ± 2.8	0.72
Cadence (rpm)	105.3 ± 8.4	103.4 ± 4.1	0.96
HR (beats.min^−1^)	171 ± 9.9	171 ± 8.0	0.94
Duration (s)	2191 ± 157.6	2181 ± 193.9	0.61
% difference (IC 90%)	−10.1 (−45 to 24.9)		
% difference positive/trivial/negative	2/85/12		
Qualitative Inference	Unclear		

*CAF* = caffeine; *PLA* = placebo.

**Figure 2:**
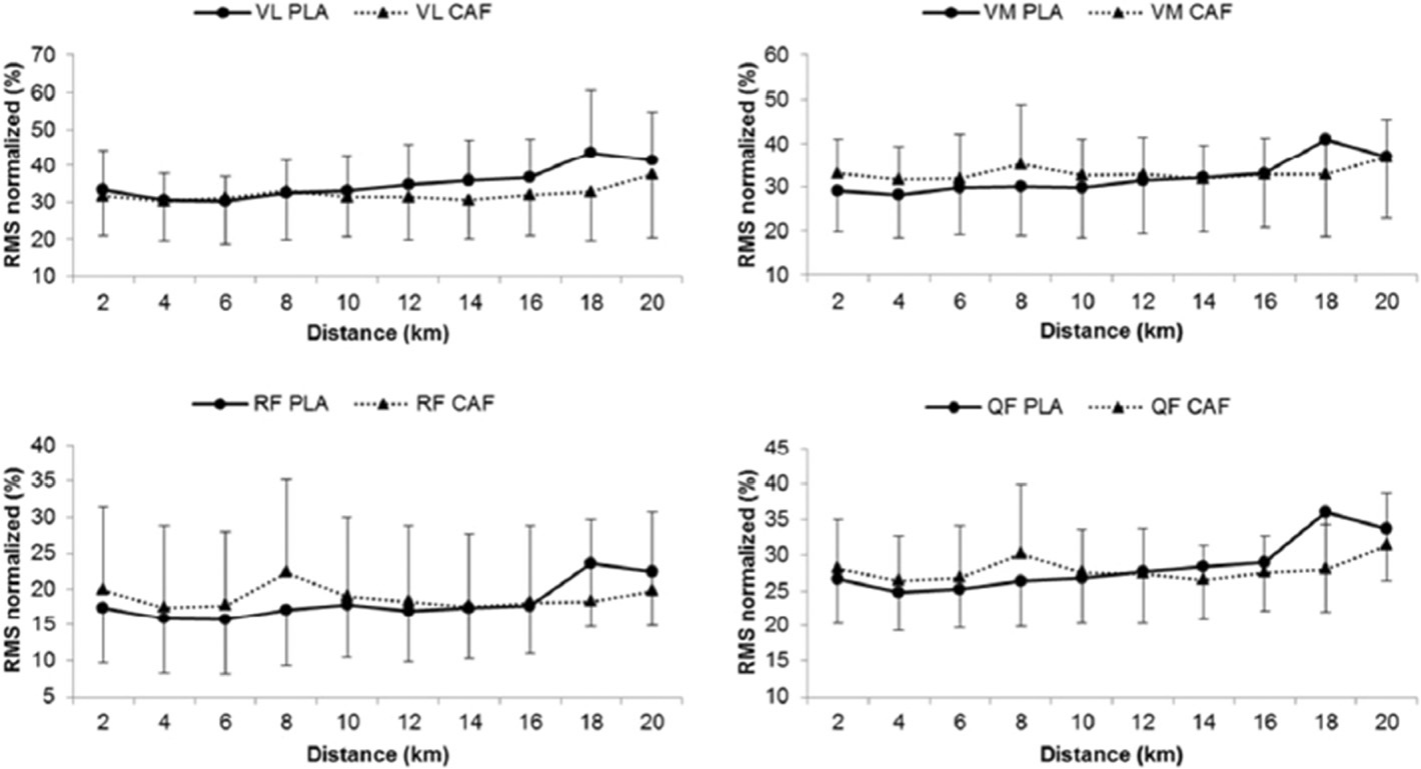
**Pattern of EMG activity of the VL, RF, VM and QF muscles during the 20-km time-trial test under the conditions CAF (n = 12) and PLA (n = 12).** No main effect or group vs. time interaction was identified (*P* > 0.05).

**Figure 3:**
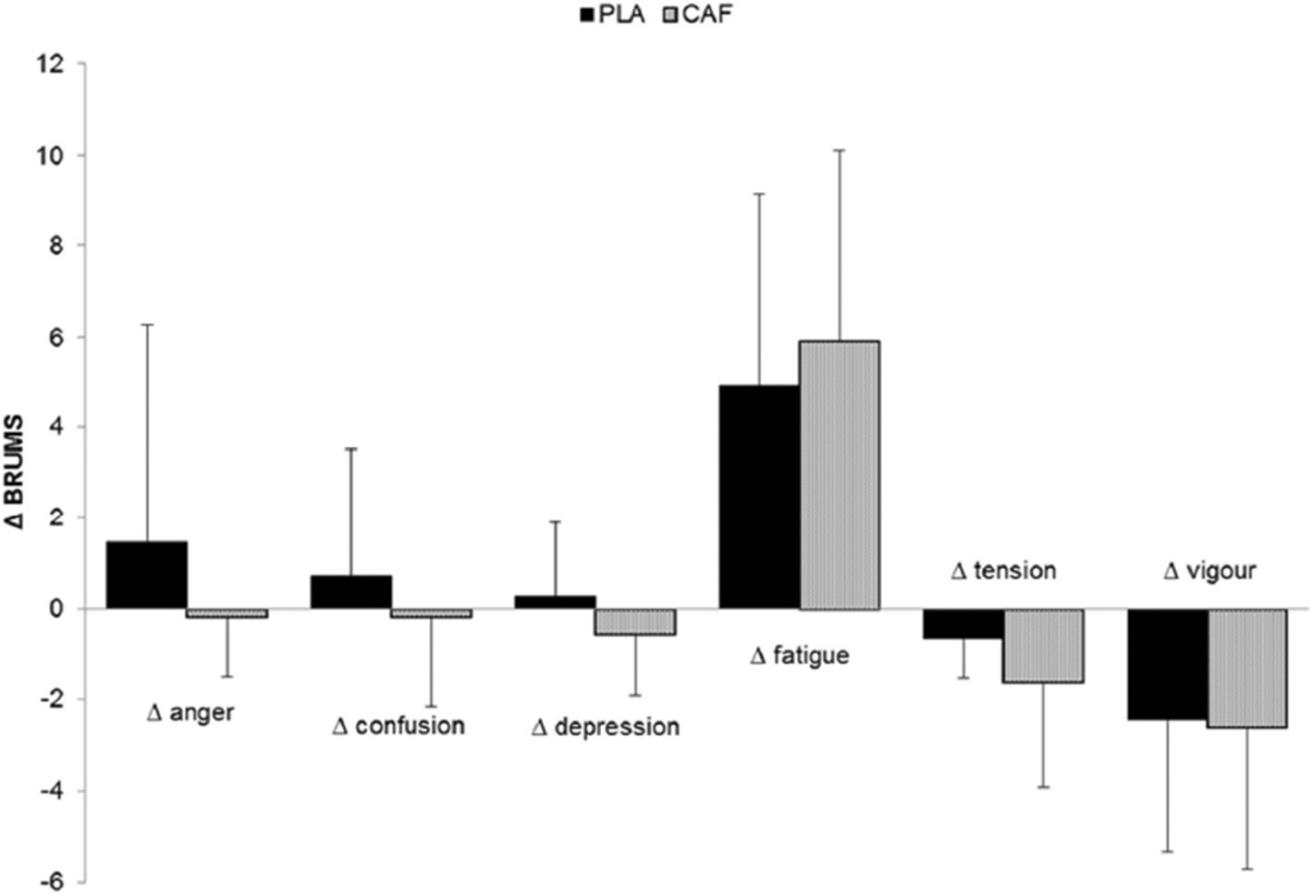
Variation delta of mood (BRUMSpost – BRUMSpre) in their various domains in the 20-km time-trial (n = 13).

The EMG pattern during the tests is presented in Figure 2. No difference was found between the two experimental conditions (PLA and CAF) for the VL, RF, VM and QF muscles. Thus, no significant group main effect or group by moment interaction was identified (*P* > 0.05). There was a progressive increase in the RPE during the test in both groups, without any statistically significant differences between them (*P* > 0.05). Only a significant distance main effect was identified for HR and RPE (*P* < 0.001). No statistically significant difference (*P* > 0.05) was detected in the RPE increase rate between groups (PLA = 0.88 points.km^−1^ vs. CAF = 0.95 points.km^−1^). Mood changes before and after the 20-km time trials are illustrated in Figure 3.

## Discussion

The main result obtained in this study was that the oral administration of 6 mg.kg^−1^ of body mass of CAF 60 min before the effort had no effect on the performance of cyclists in the 20-km time trial. The results also indicated that the use of CAF did not promote any changes in pacing strategy during the test or attenuation of RPE.

Although our results are interesting, comparisons with previous studies are really very difficult due to differences in the protocols. In a time trial study performed by McNaughton et al. [16], although the distance was similar to that used here, the authors included some uphill stretches, which made the test harder, naturally forcing their athletes to assume different pacing strategies. Additionally, their subjects ingested CAF in the form of a low-kilojoule flavored drink, and the authors did not mention whether the subjects were able to distinguish between the drink containing CAF or PLA. In another study conducted by Ivy et al. [15], CAF was used in combination with other substances (labeled as an “energy drink”) to compete a fixed amount of work on a cycle ergometer in significantly less time than after consuming a placebo. Thus, the results of these studies cannot be compared with our results.

The stimulatory effect of CAF on the central nervous system appears not only to modify the parameters of motivation, but also to attenuate RPE, enabling cyclists to sustain the discomfort caused by exercise. The magnitude of this effect has been reported to be close to 6% during constant load exercise, increasing time to exhaustion [10]. However, this effect was not observed in this study. Our results showed that RPE showed no differences when the two trial conditions were compared. The RPE increase rate verified by the slope on the regression plot for RPE values throughout the test, showed no significant differences between conditions (0.88 points.km^−1^ vs. 0.95 points.km^−1^, for PLA and CAF, respectively).

In open protocols, individuals usually must maintain a fixed work rate to exhaustion. Thus, the fact that there is no defined end prevents pacing strategy planning [14]. However, when the subject does not necessarily need to keep a fixed intensity, this allows the development of strategies during the race aiming at finishing in the shortest possible time. Therefore, investigations on CAF effect on performance in tests that mimic the actual conditions found in competitions could be more relevant and strengthen the importance of the results found.

Pacing strategy planning is centrally mediated. Due to its direct action on the nervous system, CAF should, therefore, influence and change pacing strategy during 20-km time trials. These changes should be observed by different power, speed and/or rpm behaviors during the tests. However, our results failed to show any influence of his level of CAF intake on pacing planning. This confirms the results of Hunter et al. [14], who demonstrated that CAF not only had no effect on EMG, RPE, HR and performance (time) parameters during 100-km time trials, but it also had no influence on pacing strategy. Only in the final part of the test were significant differences in pacing strategy observed when compared to the remainder of the exercise. This has already been shown in a previous study where pacing strategy varied only minimally in the last 30 s of a 30-min time trial [24].

Few studies have investigated the effect of CAF without combination with carbohydrates on medium and long time trial distances (>5 km) Bruce et al. [13] demonstrated that CAF ingestion significantly improved the performance of rowers in the first 500 of 2000 m trials. The authors suggested that CAF may act directly on subconscious brain centers responsible for pacing strategy planning during exercise [13]. On the other hand, Cohen et al. [25] showed a decrease in performance of 0.7% in a 21-km race protocol, after the subjects had ingested capsules of CAF (9 mg.kg^−1^) 60 min prior to the beginning of the exercise. In a 20-km race protocol, 60 min after the ingestion of CAF capsules (6 mg.kg^−1^), individuals improved performance in 1.7%, but this increase was not significant [26]. In this study, we found an improvement of only 0.46% (~10 s) in the performance, again not significant.

Throughout the test, EMG showed no differences between the experimental conditions and along the 20 km. Muscle activation during the tests was ~25% of the values obtained in the TV-test, with no significant changes at any time. This suggests the absence of peripheral fatigue during testing. Similarly, Hunter et al. [14] also failed to identify changes in EMG at any point along the 100 km time trial. During exercise, there is a decrease in muscular strength, and the amplitude of the EMG signal should increase to sustain the same intensity of exercise and/or stay on the task, increasing the firing rate. As a result, the amplitude of the EMG signal should be higher for the same power. But we could not confirm the absence of neuromuscular fatigue during the test as RPE gradually increased. These could be better discussed with the use of different techniques for the assessment of central and/or peripheral fatigue, such as the level of maximal voluntary activation measured by the twitch interpolation technique [27].

In the present study, the BRUMS’s scale, which is intended to allow a quick measure of mood [28], was applied immediately before and after the tests in order to verify possible changes promoted by the administration of CAF (Figure 3). We expected that CAF would modify mood variation, relieving fatigue, and/or strength symptoms, which would explain possible improvements in performance. However, no significant differences were found between the experimental conditions.

In the present study we aimed at controlling key variables previously mentioned in the literature, to generate reliable and reproducible information. Thus, some methodological precautions were taken. It is known that several factors appear to influence CAF’s potential and magnitude ergogenic effects, such as the way the substance is administered (capsules, drink, or gum), the moment the substance administered (prior and/or during exercise), whether CAF is associated with some other substances (carbohydrate) or not, fasting status, and habituation, among others [3]. In the present study, subjects were asked to avoid eating foods containing CAF 48 hours before the test to minimize the possible influence of the level of habituation on the results. However, the level of habituation to CAF and the subjects’ eating habits were not directly controlled. It has been shown that after a period of 2 to 4 days of CAF withdrawal, a tendency to potentiate the effects of CAF on the protocol until exhaustion does exist, when compared to 0 days, but without any differences between those times [29]. However, in an animal model, an increase in the number and affinity of adenosine receptors after 7 days of CAF abstinence was observed [30]. Hence, studies seeking to demonstrate the effect of a prolonged period (>7 days) of CAF abstinence on performance in humans could be of interest. In sports, it might be speculated that when habituation to CAF exists, a restriction in the consumption of this substance for a period of approximately seven days may provide gains and/or potentiate the effect of CAF. But this hypothesis has yet to be verified.

Another limitation of this study was that athletes in the present sample only participate in local competitions making it difficult to extrapolate our findings to well-trained athletes, who compete internationally. This probably explains the low power values found here compared to studies that used well-trained athletes [31]. Unfortunately, studies with tests of similar duration that demonstrated an ergogenic effect of CAF alone [16] or in combination with other substances [15] have reported no data on the average power observed during the time trial, or the maximum power in the incremental test, again making comparison difficult.

In conclusion, our results do not encourage the supplementation with CAF in a cycling time trial setting. Studies involving shorter protocols, similar to cycling events, should be tested for better understanding the use of CAF in closed-loop protocols. Furthermore, future studies should also seek to demonstrate whether CAF abstinence for longer periods could enhance performance on closed protocols and the mechanisms involved in fatigue during exercise.

## Competing interests

The authors declare that they have no competing of interests.

## Authors’ contributions

HB, LRA, MVC and ESC were significant manuscript writers; HB, LRA and ESC participated in the concept and design; HB and MVC were responsible for data acquisition; HB, LRA, MVC and ESC participated in data analysis and interpretation. All authors read and approved the final manuscript.
